# Ranking of Risk Factors Leading to Uterine Scar Defect—Systematic Online Review

**DOI:** 10.3390/jcm14134551

**Published:** 2025-06-26

**Authors:** Ionita Ducu, Bianca-Margareta Salmen, Ana-Maria Iordache, Cristiana-Elena Durdu, Roxana Elena Bohiltea

**Affiliations:** 1 Doctoral School, “Carol Davila” University of Medicine and Pharmacy, Dionisie Lupu Str., Nr 37, Sector 2, 020021 Bucharest, Romania; ionita.ducu@drd.umfcd.ro (I.D.); bianca-margareta.mihai@drd.umfcd.ro (B.-M.S.); 2 Life Memorial Hospital, Calea Griviței Str., Nr 365, Sector 1, 010719 Bucharest, Romania; 3 Filantropia Clinical Hospital of Obstetrics and Gynecology, Ion Mihalache Blv., Nr 11–13, Sector 1, 011171 București, Romania; roxana.bohiltea@umfcd.ro; 4 Optospintronics Department, National Institute for Optoelectronics-INOE 2000, Atomistilor Str., Nr 409, 077125 Magurele, Romania; 5 Department of Obstetrics and Gynecology, University of Medicine and Pharmacy “Carol Davila”, Eroii Sanitari Bvd., No. 8, Sector 5, 020021 Bucharest, Romania

**Keywords:** uterine scar defect, risk factors, single- vs. double-layer suture, adhesions, infections

## Abstract

**Background**: Cesarean deliveries have increased in recent years worldwide. This increase translates into an escalation of obstetrical complications that could lead to permanent injuries. Comprehensive knowledge of the risk factors for uterine scar defects and their probability factor could guide gynecologists towards decreasing the percentage of scar defects and reducing the morbidity produced by a scarred uterus. **Methods**: A review of the literature published in the last 10 years produced a number of 80,085 articles, from which we screened 147 articles and selected 11 recently published papers, attempting to rank the most frequently described risk factors in terms of probability. A total number of 11,349 patients who underwent CS were included from the 11 studies. **Results**: Cesarean scar defects developed in 19.42% of cases; our results showed that the highest probability was given by single-layer suture, with gestational diabetes being the main patient-related risk factor for scar defects. A definitive ranking of the risks is difficult to assess, because different studies focus on certain risks and most of the relevant data are missing or are omitted. **Conclusions**: In this study, we investigate the most common risk factors that give rise to the development of cesarian scar defects, conducting a ranking of these risks from the most probable to the least important, in order to facilitate informed decision making for providers.

## 1. Introduction

Starting from 1882, cesarean deliveries are a life-saving intervention which increase the survival of both mother and child [1,2,3,4]. However, among the complications that arise from cesarian sections (CSs), cesarian scar defect is the most prevalent [5,6]. Also called “isthmocele”, this defect appears following the process of poor healing of the myometrium. The exact causes of faulty healing of the uterine scar are unknown, but diabetes and obesity have been presumed to have negative effects due to chronic inflammation, insulin resistance, and hyperglycemia [5]. Normally, during closure of the incision, the margins of the wound come in close contact and, during the inflammation stage, begin migration, matrix remodeling, and tissue regeneration. Debras et al. [7] concluded that the inflammation phase is decisive for the healing process because it leads to the organization and maturation of collagen, which, in turn, can influence the biomechanical properties of the uterus. Gezer et al. [8] proposed a non-endometrial suturing technique to reduce the development of cesarean scar defects (CSDs). The concept behind the technique is to allow for a natural alignment of the myometrial edges, thus reducing ischemia. Other authors focus on other surgical techniques, such as horizontal mattress [9], far-far, near-near [10], or double-layered purse string [11,12], assessing their superior characteristics compared to traditional single-layer or double-layer suture techniques [13]. A useful comparison of different strategies for uterine closure, showing their different advantages and disadvantages, was published by Seyedi-Moghadam et al. [14].

Klein Meuleman et al. affirmed that 60% of cesarean deliveries produce CSDs [15].

Two recent Delphi studies [15,16] found that the main symptoms which can indicate the presence of a scar defect are post-menstrual spotting, abnormal blood loss, dyspareunia, abnormal vaginal discharge, chronic pelvic pain, and unexplained infertility. These symptoms can be an indicator of further gynecological and obstetrical complications, such as cesarean scar pregnancy, uterine dehiscence, uterine rupture, and placenta accreta spectrum. Diagnostics of the CSD are typically performed with transvaginal sonography or contrast-enhanced ultrasonography [5]. These studies have led to the elaboration of an encompassing definition of scar defects in the UOG journal [17] as follows: “niche should be defined as an indentation at the site of the CS scar with a depth of at least 2 mm”. The appropriate method to view and measure a CSD should begin in the midsagittal plane, as to obtain a good visualization of the cervical canal, then the transvaginal probe should be moved laterally to both sides. Measurement should be based only on the myometrium, and contrast sonography will give an added value in patients with uterine scar defects [16]. The authors also subclassified scar defects as follows: “(1) simple niche, (2) simple niche with one branch or (3) complex niche (with more than one branch). A branch is a thinner part of the main niche, directed toward the serosa and with a smaller width than that of the main niche. Niche measurements that should be performed in basic evaluation (niche length, depth, residual myometrium thickness, adjacent myometrium thickness, and width)” [17].

Medical providers usually focus on the following seven risk factors that are thought to determine the development of a cesarian scar defect following CS [18]: (1) gestational diabetes; (2) an advanced maternal body mass index; (3) multiple caesarian deliveries—CDs; (4) a longer duration of active labor before performing CD; (5) the presence of adhesions; (6) infections; and (7) the type of suture used when closing the uterus. However, a recent study identified a full series of risk factors divided into the following three categories: patient-related, labor-related, and surgery-related (Table 1) [19].

None of the studies screened by the authors give a clear picture of the ranking of these risks: is gestational diabetes the major potential risk factor, or is it the type of suture the surgeon chooses? How many cesarian deliveries are too many, and how important is the presence of infections and adhesions in the development of a cesarean scar defect? Several studies have demonstrated that an advanced maternal body mass, gestational diabetes, and multiple CDs are associated with impaired healing of the uterine incision [5,20].

In this study, we aim to investigate the most common risk factors that give rise to the development of cesarian scar defects and to attempt a ranking of these risks from the most probable to the least important, in order to facilitate informed decision making for providers.

## 2. Materials and Methods

**Figure 1:**
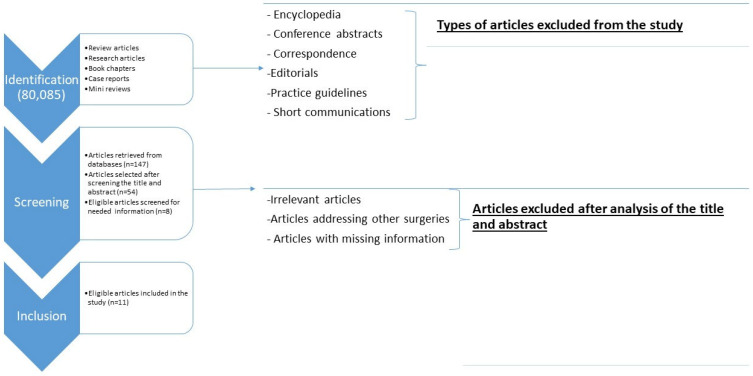
Illustration of the selection process of the articles included in this study.

This study is a systematic online review of all the known risk factors presented in the literature, and we focus on the descriptive analysis for the major risk factors. We employed the search engines ScienceDirect, PubMed, UpToDate, and Scopus to screen over 100 articles dealing with different risk factors that lead to the formation of a defect in a cesarian scar. Our search focused on “uterine scar defect”, “niche defect”, “uterine niche defect”, “isthmocele”, “isthmocele risk factor”, “pathogenesis of caesarean scar”, and “pathogenesis of isthmocele”. The inclusion criteria were (1) review articles, research articles, book chapters, mini reviews, or case studies dealing with pregnant women undergoing cesarean surgery (elective/emergency) and (2) a comparison of different risk factors that induce the development of a cesarean scar defect. The exclusion criteria were encyclopedia, conference abstracts (unavailability of the full text), correspondence, editorials (due to bias errors), practice guidelines, and short communications. The selection process is shown in Figure 1 and the Prisma flowdiagram is provided in the Supplementary Files (Figure S1)

In order to obtain a wider view of all the risk factors, we did not apply restriction to publication year or language (we included one article in French [21]). Teenage pregnancies were also excluded. To limit duplicate records and to easily manage the references, we used EndNote software (ver 2021). Also, we focused on studies that defined the scar defect as the niche which opens in the uterus and has a depth of more than 2 mm, in order to homogenize the measurements. Data selection and extraction, as well as interpretation, were independently reviewed by two other authors to limit bias.

This trial was registered in the Prospero database, and is available at https://www.crd.york.ac.uk/prospero/ (trial registration: CRD42024533262), accessed on 7 April 2024, published on 19 April 2024.

To the best of our knowledge, no other article has introduced the risk coefficient in their analysis. This is why, for the first time, we introduce the risk coefficient, defined as the conditional probability [22] between probability and *p*-value and calculated with the following formula:

(1) $$risk\;coefficient=\frac{P}{p‐value}$$

where

(2) $$\mathrm{probability}:\;P=\frac{no\;of\;favorable\;cases}{all\;cases\;posible}$$

where no of favorable cases is the number of cases where a CSD is present.

We ranked the risks from the most probable to the least possible as a function of the risk coefficient (rather than probability).

Following screening, we selected 11 papers published in the last 10 years (distributed over the years as follows: 1 paper in 2017, 1 paper in 2018, 2 papers in 2019, 1 paper in 2021, 1 paper in 2022, 3 papers in 2023, and 2 papers in 2024) containing most of the information we required. The reason we focused on the last 10 years is the fact that these recent studies tend to provide more detailed parameters that we can use to perform further analysis. By tabulating and performing statistical analysis with MICROSOFT EXCEL 2021 and Origin 2021 software, we calculated the probability, expressed as a percentage (%), mean, and standard deviation of the data (descriptive analysis), as well as odds ratios (ORs) and relative 95% confidence intervals, using the inverse variance method. *p* values of < 0.05 were considered significant. None of the papers presented all the information needed.

## 3. Results

Our study included a total number of 11,349 patients who underwent CS (from the 11 studies), and 2204 out of those, equivalent to 19.42%, developed a cesarean scar defect. Table 2 shows an overview of the studies selected for the analysis. From these selected studies, only seven compared the type of suture used (single-layer vs. double-layer).

Since the studies could not provide all the parameters needed, we selected six risk factors to be analyzed from patient-related risks and six risk factors from surgery-related risks. Table 3 shows the statistical analysis we performed for the selected studies. It shows that three risks factors had a probability below 0.05, while nine risk factors had probabilities above this value. The three risk factors with *p*-values of <0.05 (which is statistically relevant) were gestational diabetes, previous CS, and cervical dilatations of ≥5 cm. However, this limitation is an impediment for our aim, which is to rank the risk factors in a logical order.

By introducing the conditional probability as risk coefficient, we were able to perform the ranking of these six most important risk factors (patient- and surgery-related risks). Our results show that gestational diabetes is a major risk factor, together with previous CS. Compared to these, we observe that infections and advanced maternal BMI are not serious risks in the development of cesarean scar defects. Moreover, surgery-related risks showed even lower values, with double-layer suture being placed last on the risk scale. The data is shown in Table 4.

## 4. Discussion

Our results, in terms of what type of suture better suits closure of the uterus, are in contradiction with the results of Roberge et al. [31,32], but are in line with the studies of Tang et al. [23] and Sevket [33], because the precise pathways that produce a cesarean scar defect are not fully known. Our study concludes that a single-layer suture has a higher probability of developing into a CSD than a double-layer suture. A possible explanation is that double-layer closure promotes slower but steadier healing of the uterus [23]. More pertinent studies dealing with repair sutures [34,35] contradict this explanation and propose that double-layer sutures reduce the risks of hematoma and scar dehiscence. However, in our view, a better explanation for the discrepancy of the results is the different definitions of cesarean scar defect used by different research groups [28]. In 2021, a large, randomized control trial that compared single- vs. double-layer non-locking sutures in women undergoing their first CS, found that the prevalence of scar defects (defined as a 2 mm defect in the thickness of the myometrium) at three months was 68.9% for single-layer closure compared to 73.6% for double-layer closure [36].

Gestational diabetes was the most important risk factor revealed in our study. It also had the highest probability. Only a limited number of studies have dealt with the correspondence between gestational diabetes and scar defects. It is important to distinguish gestational diabetes vs. type 1 diabetes: gestational diabetes is caused by the interference of pregnancy hormones with insulin and is usually diagnosed at >20 weeks of pregnancy, while type 1 diabetes is determined by the inefficiency of insulin (its diagnosis is given before 20 weeks of gestation). For the purpose of wound healing and the limitation of the occurrence of cesarean scar defects, the most important factor is achieving glycemic control through initiating the correct treatment for diabetes [37]. This is especially true for pregnant patients, because uncontrolled gestational diabetes can lead to fetal development impairments, perinatal complications, and can affect women’s predisposition to future diabetic pathologies [38,39].

Further down the scale, previous cesarian deliveries had a 11.28 risk coefficient. Our results for multiple caesarean sections are similar to other studies [40,41,42,43]. These results showed that women without a previous CD had a 35% chance of developing a CSD, while after multiple caesarian deliveries, the risk of developing scar defects increased to 63% (after the first CD), 76% (after the second CD), and 88% (after the third) [7]. The reason we did not include any of these studies in our analysis is the fact that they either used the same suture technique for the entire cohort of patients or that they did not stipulate the type of suture used for wound closure—missing parameters. Moreover, the study performed by Debras et al. [7] focused exclusively on histological analysis of the uterine scar area in order to explain the occurrence of scar defects with the help of model studies in both humans and animals.

Infections after caesarian deliveries also play a role in the development of a CSD. Our results show a low-risk coefficient (of 2.08) and are based on studies from the literature, where infections negatively influence the healing of the scar by reducing blood perfusion and oxygenation [19]. By administering antibiotics, bacteria are killed off, infection is stopped (or slowed), and inflammation is reduced [44]. Reducing inflammation will accelerate wound repair by following one of the following two pathways: (1) increasing the production of fibroblasts and other extracellular matrix components or (2) the re-organization of collagen fibers, neovascularization, leading to the formation of granulation tissue. Both pathways increase tissue resistance, but decrease elasticity [45,46].

Concerning advanced maternal BMI, our results show a lower probability of it inducing or actively participating in the formation of a cesarean scar defect, most likely because our studies were based on patients with a mean BMI (during CD) of 28.5 kg/m^2^. Only 25% of the patients in our study had a BMI over 30 kg/m^2^. Still, Verberkt et al. [19] showed that for every additional unit of BMI, the risk of developing a CSD is raised by 6%. However, the risks associated with obesity are primarily correlated with hypertension [47] rather than wound healing.

Table 5 shows a comparison of our data with other studies in the literature. Although our manuscript shows that only 19.42% of the total patients developed a cesarean scar defect, which is a lower value when compared with other studies, this is due to the fact that studies usually focus on a certain risk (or just several risk factors) and a very specific group of patients. Table 5 shows this heterogeneity of data in the literature. In this manuscript, we tried to rank 12 risk factors (Tabel 3), but were able to perform ranking for only 6 (Table 4) due to the lack of parameters provided by the selected studies. Another finding was the fact that each author defined scar defects and the risk factors by using different analytical tools, e.g., the authors created different groups for each measurement of the scar defect parameters (ranging from thinning area, depth of the defect, thickness of the myometrium, etc.) [48]. This explains why we observed variability (up to 30–70% differences between the results) in the results of the studies we analyzed, and also explains why studies researching the same risk factors reach different conclusions. This lack of standardization between the definitions of risk factors could be the reason for the discrepancy in the studies.

The strengths of this study include its inclusion of recent papers, i.e., published in the past 10 years, with a cumulative number of patients of 11,349. We focused on studies that defined scar defects as a niche that opens in the uterus and has a depth of more than 2 mm as supported by the Delphi studies, in order to homogenize the measurements. Data selection and extraction, as well as interpretation, were independently reviewed by two other authors to limit bias.

The limitations of this study include the selected studies varying in terms of methodology, population, and outcomes, which could lead to errors in our analysis. The heterogeneity of time intervals for the detection of cesarean scar defects in the included studies makes it difficult to maintain a stable analysis. An expansion of the relevant studies is still needed, particularly to include articles which have all the relevant information to perform the ranking analysis. This limitation is reported by many researchers, because it is difficult to gather articles that have all the information required for systematic reviews. Another limitation refers to the fact that relationships among individual risk factors were not considered (we considered individual risk factors as separate factors).

## 5. Conclusions

A definitive ranking of risks is difficult to assess, because different studies focus on certain risks and most of the relevant data are missing or are omitted. In this article, we analyzed and performed a ranking of the main six risk factors using the definition established by the Delphi study. The main risk factor was calculated to be diabetes, followed by previous CS and infections, while advanced maternal BMI, single-layer suture, and double-layer suture had the lowest risk coefficients. However, not all the authors in the studies we analyzed used the same definitions as we did. This heterogeneity is a true bottleneck that prevents us from reaching a clear result in ranking the risks. Taking all of this into consideration, we propose a standardization in the definition of each risk factor connected to a specific standardization of gestational, technical, and therapeutic parameters in order to perform a clear assessment and ranking of the demonstrated risk factors.

## Figures and Tables

**Table 1:** All known risk factors related to the development of cesarean scar defects, according to [19].

Patient-Related Risk Factors	Labor-Related Risk Factors	Surgery-Related Risk Factors (Closure Technique)
Multiple CS	Timing of CS	Suture material
BMI	Duration of contraction	Unlocked vs. locked
Maternal age	Cervical effacement	Single vs. double layer
Position of uterus	Thickness of lower uterine segment	Inclusion vs. exclusion of the endometrial layer
Gestational diabetes	Level of the uterus incision	Distance from the scar to the external cervical ostium
Gestational age and interval between CD		
Neonatal birth weight		
Pre-eclampsia		
Smoking		

**Table 2:** Overview of the studies selected for analysis.

Ref.	Type of Study	Total No *	Cesarean Scar Defect **	Type of Suture		Identified Risk Factors
				Single-Layer	Double-Layer	
[5]	Cohort study	371	169	1	370	Gestational diabetes Previous cesarean delivery Advanced maternal body mass index (mean of 32.3 kg/m^2^) Longer duration of active labor (mean of 16.2 h) *** Infection RMT < 3 mm
[20]	Cross-sectional	297	194	194	0	Advanced maternal body mass index (mean of 26.8 kg/m^2^) Prolonged duration of active labor (mean of 16.2 h) Diabetes
[8]	Randomized controlled trial	159	36	159	0	BMI (mean of 28.5 kg/m^2^) *** Surgical site infections
[23]	Case–control study	567	189	96	93	Previous cesarean delivery *** Infections
[24]	Prospective clinical trial	250	33	0	250	BMI (mean of 35 kg/m^2^) Prolonged labor *** Infection
[25]	Prospective research	32	1	-	-	Previous cesarean delivery BMI (mean of 25 kg/m^2^) Failure to progress
[26]	Prospective, randomized clinical study	126	72	24	48	BMI (mean of 27 kg/m^2^)
[27]	Clinical trial	74	31	20	11	BMI (mean of 28 kg/m^2^)
[28]	Prospective randomized study	324	251	149	175	BMI (mean of 23 kg/m^2^) Previous surgery on the uterus Gestational diabetes *** Infections
[29]	Systematic review	8799	1107	556	541	BMI (mean of 25.6 kg/m^2^) Prior CS *** Infections
[30]	Systematic review	350	121	164	186	Prior CS

* Total number of patients enrolled in the study; ** number of patients diagnosed with cesarean scar defect; *** in the infections category, we included chorioamnionitis, postpartum wound infections, and endometritis.

**Table 3:** Results of the statistical analysis for the major risk factors assessed in this paper.

Type of Risk	Risk Factors	Cesarean Scar Defect Absent (−)	Cesarean Scar Defect Present (+)	OR	95% CI	*p*-Value
Patient-related risks	Gestational diabetes	49	74	1.33	0.89; 1.99	0.04363
	Infections	81	92	1.76	1.28; 2.42	0.26
	Previous CS	533	463	1.74	1.31; 2.31	0.0412
	Advanced maternal BMI	20	26	0.62	0.36; 1.05	0.33
	Uterine position at ultrasound					
	Anteroversion	397	394	1.49	1.21; 1.85	0.24
	Retroversion	161	250	2.41	1.91; 3.05	0.37
	Cervical dilatation at CS (cm)					
	0	168	100	0.187	0.13; 0.26	0.34
	1→4	62	202	3.18	2.26; 4.47	0.23
	≥5	45	118	1.99	1.36; 2.93	0.002
Surgery-related risks	Single-layer suture	284	370	1.7	1.40; 2.05	0.38
	Double-layer suture	1000	633	0.43	0.35; 0.51	0.34
	Locked suture	55	127	9.08	6.15; 13.1	0.33
	Unlocked suture	641	513	0.1	0.05; 0.18	0.43
	RMT ≤ 3 mm	0	322	-	-	-
	RMT >3 mm	282	387	1.75	1.42; 2.15	0.48

**Table 4:** Output of the data analysis for the selected studies, showing our ranking of the risk factors compared with the development of CSD.

Risk Factor	Probability (P)	*p*-Value	Risk Coefficient (P/*p*-Value)	OR	Ranking	Highest Risk
Gestational diabetes	0.60	0.04	13.78	1.33	I	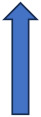
Previous CS	0.46	0.04	11.28	1.74	II	
Infections	0.53	0.26	2.04	1.76	III	
Advanced maternal BMI	0.56	0.33	1.7	0.62	IV	
Single-layer suture	0.56	0.38	1.48	1.7	V	
Double-layer suture	0.38	0.34	1.14	0.43	VI	Lowest risk

**Table 5:** Comparison of our data with other data from the literature.

Ref.	Study Type	No of Patients	Evaluation Method (Single vs. Double)	Outcome (Findings)
[49]	Prospective cross-sectional study	280	Saline infusion sonography (SIS)	No statistically significant difference was found in the size of the uterine scar defect between single- and double-layer closure
[50]	Prospective longitudinal study	43	Transvaginal sonography	The cut-off value for the CS scar thickness was 3.0 mm Ultrasound assessment of the quality of the CS scar healing process is already feasible at 6 weeks after CS
[10]	Prospective, randomized, controlled study	138	Vaginal B-ultrasound	Uterine closure using the far-far-near-near technique is beneficial for providing protection from cesarean scar defect formation This uterine closure ensures sufficient RMT compared with single-layer locked closure technique
[51]	Prospective study	765	N/A	A locked uterine closure performed in caudal direction is the optimal closure technique.
[52]	Prospective cohort study	267	Saline contrast sonohysterography	Single-layer closure is associated with an increased risk of larger cesarean scar defects
Our study	Statistical analysis	11,349	Raking based on risk coefficient	Gestational diabetes is the main risk factor, followed by previous CS, infections, and advanced maternal BMI. Single-layer suture and double-layer suture have the lowest risk coefficients

## Supplementary Materials

The following supporting information can be downloaded at: https://www.mdpi.com/article/10.3390/jcm14134551/s1.
