# Supplementary material for: Ranking of Risk Factors Leading to Uterine Scar Defect—Systematic Online Review
Source: J Clin Med. 2025 Jun 26;14(13):4551. doi: 10.3390/jcm14134551 (PMC12249996; doi:10.3390/jcm14134551)
Supplement: Supplementary file 1 [file jcm-14-04551-s001.zip › jcm-3638290-supplementary.pdf]

Systematic Review

Ranking of Risk Factors Leading to Uterine Scar Defect—Systematic Online Review

Ionita Ducu [1,2], Bianca-Margareta Salmen [1,3], Ana-Maria Iordache [4], Cristiana-Elena Durdu [1,3,*] and Roxana Elena Bohiltea [3,5]

[1] Doctoral School, "Carol Davila" University of Medicine and Pharmacy Bucharest, Dionisie Lupu Str., Nr 37, Sector 2, 020021 Bucharest, Romania; ionita.ducu@drd.umfcd.ro (I.D.); bianca-margareta.mihai@drd.umfcd.ro (B.-M.S.)
[2] Life Memorial Hospital, Calea Griviței Str., Nr 365, Sector 1, 010719 Bucharest, Romania
[3] Filantropia Clinical Hospital of Obstetrics and Gynecology, Ion Mihalache Blv., Nr 11-13, Sector 1, 011171 București, Romania; roxana.bohiltea@umfcd.ro
[4] Optospintronics Department, National Institute for Optoelectronics-INOE 2000, Atomistilor Str., Nr 409, 077125 Magurele, Romania; ana.iordache@inoe.ro
[5] Department of Obstetrics and Gynecology, University of Medicine and Pharmacy "Carol Davila" Bucharest, Eroii Sanitari Bvd., no. 8, Sector 5, 020021 Bucharest, Romania
* Correspondence: cristiana-elena.durdu@drd.umfcd.ro

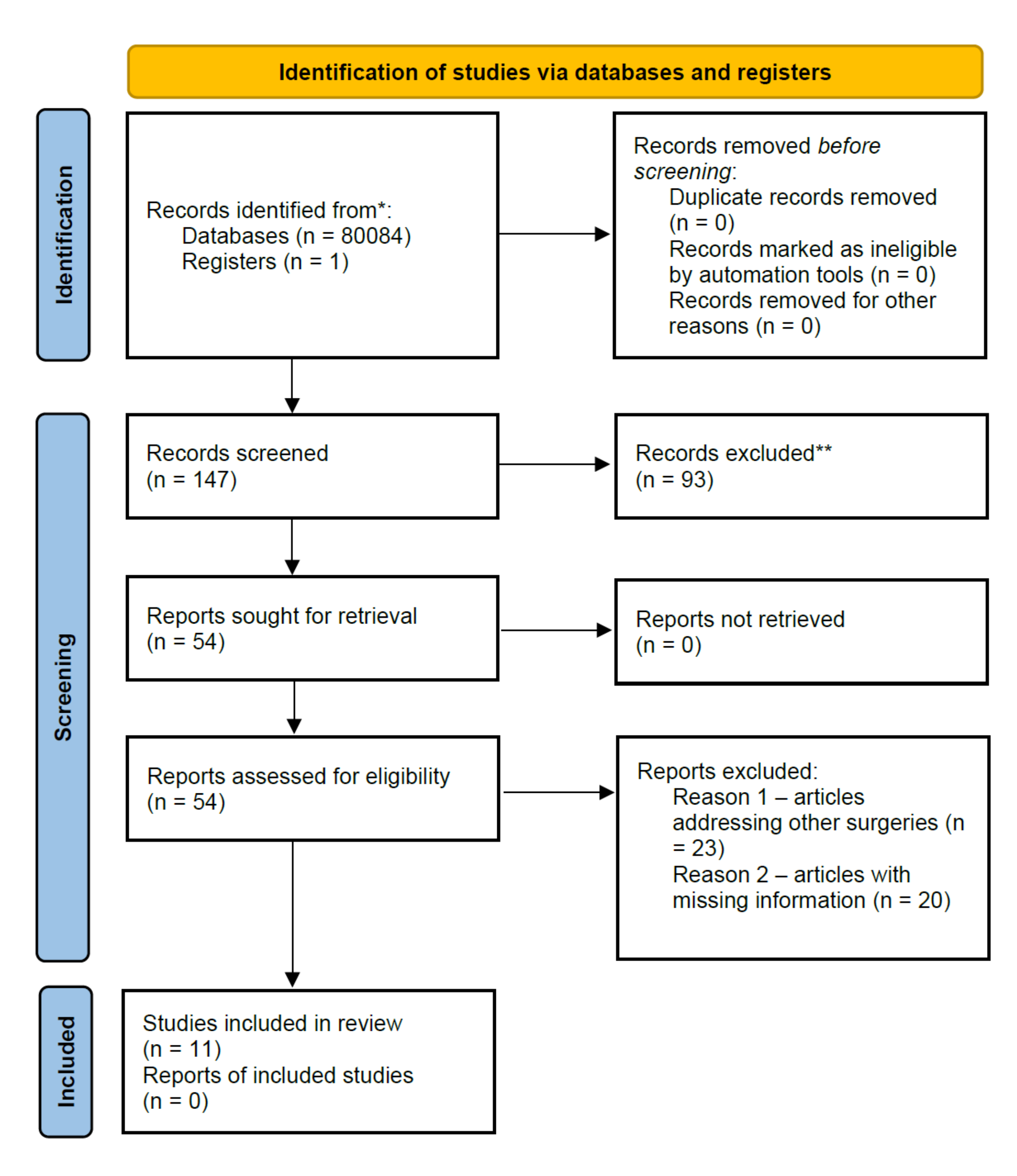

Figure S1: PRISMA 2020 flow diagram for new systematic reviews which included searches of databases and registers only.
